# Time to treatment-seeking by caretakers of children under-five with diarrhea and associated factors in Uganda: a multilevel proportional hazards analysis

**DOI:** 10.1186/s12887-024-04879-9

**Published:** 2024-06-22

**Authors:** Sula Tumusiime, John Bosco Asimwe, Leonard Atuhaire, Ronald Wasswa, Dick Nsimbe, Brian Kayera

**Affiliations:** Department of Statistical Methods and Actual Science, School of Statistics and Planning, Makerere University, P.O.Box 7062, Kampala, Uganda

**Keywords:** Childhood diarrhea, Caretakers, Treatment seeking, Multilevel-proportional-hazards, Uganda

## Abstract

**Background:**

Diarrhea is considered to be one of the major public health concerns in developing countries. It has a detrimental impact, reflecting one of the highest child mortality rates globally, especially in Sub-Saharan Africa, where 2 out of every 10 children in Uganda under the age of five die. The objective of this study was to investigate the factors associated with time to treatment seeking by caretakers of children under-five with Diarrhea in Uganda.

**Method:**

DOVE dataset of 745 caretakers in a prospective and retrospective incidence-based study using multi-stage sampling design was used in the assessment. The analysis was done using a time-to-event approach using life tables, Kaplan Meier survival analysis and multilevel proportional hazards model.

**Results:**

Kaplan-Meier survival analysis indicated the median time to seeking treatment among 745 caretakers of children under-Five after onset of diarrhea was 2 days. The multi-level proportional hazards model of a Weibull distribution showed that the estimated frailty variance was 0.13, indicating heterogeneity of treatment seeking time by caretakers of under-five children with diarrhea across regions in Uganda. Significant factors found to influence time to treatment-seeking by caretakers of children under-five with diarrhea were, male children (HR = 0.82; 95% CI = 0.71–0.95, *p* = 0.010), belonging to richest wealth quintile (HR = 1.37; 95% CI = 1.05–1.78, *p* = 0.022), and residing more than 5 km away from a health facility (HR = 0.68; 95% CI = 0.56–0.84, *p* = 0.000).

**Conclusions:**

There are delays in seeking diarrhea treatment in Uganda because two days are enough to claim a life after dehydration.The policymakers should pay attention to formulate effective intervention to sensitize caregivers on the importance of early treatment-seeking behavior to avoid severe malnutrition caused by diarrhea. Community awareness program should also be encouraged particularly in areas of more than 5 km from the health facility to make people aware of the necessity to take prompt action to seek care in the early stage.

## Background

Diarrheal diseases contribute to 1 in 9 child deaths globally, making it the second leading cause of mortality among children under 5 years old [1]. According to Liu et al. (2015), of the 6.3 million deaths recorded in children under 5 worldwide in 2013, 10% were linked to diarrhea, resulting in a daily toll of up to 2,195 children, surpassing the combined fatalities from AIDS, malaria, and measles [2]. The majority of these childhood deaths are concentrated in South Asia and Africa [3]. The diarrhea mortality rate per 100,000 children exhibited a significant decline of 69.6% from 1990 to 2017.Contributors to the decline in diarrhea mortality rates include improvements in sanitation, reduction in childhood wasting, and increased availability and use of oral rehydration solution (ORS) [4]. Despite the effectiveness of ORS in reducing diarrhea-related deaths, challenges persist in ensuring widespread access and utilization of this life-saving intervention. Financial constraints often hinder access to ORS and other essential healthcare services, exacerbating the burden of diarrhea morbidity and mortality, particularly among economically disadvantaged populations.

Rotavirus stands as the primary contributor to severe childhood diarrhea, causing an estimated 192,700 deaths annually, with approximately 50% of these fatalities concentrated in the World Health Organization Africa Region [5]. The World Health Organization (WHO) further projects that 7.3% of deaths among children under 5 in Uganda and 6.4% in Kenya can be attributed to rotavirus [6]. The introduction of rotavirus vaccines emerges as a pivotal strategy to significantly mitigate childhood morbidity and mortality. Post-introduction studies conducted in the USA and other regions have underscored the positive impact of vaccine implementation, particularly in reducing the disease burden, notably through a decline in rotavirus-related hospitalizations. In Uganda, spanning from 2016 to 2033, the adoption of the rotavirus vaccine holds the potential to prevent approximately 4 million cases of rotavirus diarrhea and avert 70,236 deaths [6].

In Uganda, studies and reports on child morbidity and mortality have consistently shown that diarrhea is a major public health concern [7]. In 2008, it was reported that 16% of all deaths among children under 5 in Uganda were attributed to diarrhea [8]. The lack of accessible and adequate health facilities to handle diarrhea cases has contributed to a significant number of child deaths [9]. According to the 2016 Uganda Demographic and Health Survey, 23% of children in Uganda had experienced diarrhea in the two weeks preceding the national survey [10]. It’s against this background, our study aimed to investigate factors associated with the time to seek treatment among children under five with diarrhea in Uganda.

## Methods

This study used secondary data from the DOVE study conducted by Makerere University School of Public Health in partnership with Johns Hopkins School of Public Health. The (2017–2018) DOVE study used a prospective and retrospective incidence-based study. A multi-stage sampling design was used to reach health facility and simple random sampling technique was used to select 48 health centers. Four districts were selected from 4 regions (Northern, Eastern, and Western & Central), 12 health facilities were then selected from each district bringing the total number of health facilities to 48. A sample of 15 caretakers of children under 5 years with diarrhea in Uganda was then selected from each health facility, bringing the total number of caretakers who were interviewed to 745 for both the patient caretaker exit survey and the patient caretaker follow-up survey. In order to ensure that data collected was geographically representative of the country and logically feasible, Kampala district from Central region, Gulu district from Northern region, Mbarara district from Western region and Jinja district from Eastern region. Facilities in each selected district were selected across all levels to ensure representativeness of hospitals, HCIV, HCIII and HCII. Health facilities from all levels were selected to appropriately represent rural and urban locations. Representativeness of type of ownership of facilities including private for profit, private not for a profit and public facility was also accounted for.

### Measures of outcome

The dependent variable was time to treatment-seeking by caretakers of children under five with diarrhea. The period was measured from time of onset of diarrhea up to the time the caretaker sought treatment in days.

### Measures of explanatory variables

The independent variables were socio-economic and structural characteristics of caretakers and children below the age of five years with diarrhea. The socio economic factors include the age of the child categorized as < 6 months, 6–11, 12–23, and > 24 months; the gender of the child indicated as female and male; the age of the caretaker recorded as 17–26 years, 27–36, 37–46, and > = 47 years; the gender of the caretaker recorded as female and male; the education level of the caretaker categorized as no education, primary level, secondary level, higher/tertiary level; marital status recorded as single, married, and others; Region categorized as Northern, Eastern, Western, and Central region; occupation coded as agriculture, business, formal employment, and others and Wealth quintile recorded as poorest, poor, medium, rich, and richest. The wealth quintile status of each household was defined based on asset scores generated through a principle component analysis (PCA) approach. The PCA considered the ownership of durable assets in the households: the households’ dwelling characteristics (e.g., wall, roof and or materials, water and sanitation facilities, and utilities) and durable goods (e.g., radio and television). Based on their asset score ranking, the households were divided into asset quintiles indicating the wealth status of the household. The structural factors include health facility indicated as government and private; and distance to health facility (km) categorized as (0–5)km and > 5 km.

### Statistical analysis

A survival data analysis approach was adopted in the investigation. Prior to the analysis, a survival variable was generated. A survival variable represented those who sought treatment and all observations were coded 1 since there was no censoring problem. The analysis was carried out at three stages: First, summary statistics, Kaplan Meier and life table was adopted for describing the probability of seeking treatment [11]. Secondly, differentials in time to treatment seeking by socio-economic and structural characteristics was assessed using the Log-rank test. Associations was established at 5% level of significance using the general format for the Log-rank test statistics for categorical variables. Third, the influence of socio-economic factors and structural factors on time to seeking treatment was assessed using multilevel proportional hazards model. The motivation for the frailty model was because of time to event clustered data with regions as clusters.

$${h_{ij}}(t|X) = {h_o}(t)exp\,(X_{ij}^T\beta + Z_{ij}^T{b_j})$$

Where ${h}_{ij}\left(t\right|X)$ is the survival time or the probability of seeking treatment of the ${j}^{th}$ patient in the ${i}^{th}$ cluster (Regions); ${h}_{o}\left(t\right)$ is the baseline hazard function of either a standard parametric such as the Exponential, Weibull or Gompertz distributions or more general spline based approach; ${X}_{ij}^{T}\beta$ are the fixed effects and ${Z}_{ij}^{T}{b}_{j}$ are random effects. The random effects follow a multivariate normal distribution, with ${b}_{j}$∼$N(0,\sum$). The Akaike Information Criterion (AIC) was used to obtain the best model. This is a statistic that is used when comparing the viability of different parametric models. For a set of models, the one with lower value of **AIC**, suggests a better model.

## Results

An analysis of the pattern of survival time was conducted by grouping them into overlapping intervals of 2 days. Table 1 reveals that out of 745 caretakers, approximately 34% (250) sought treatment within (0–2) days, while 66% (495) delayed in seeking treatment.

**Table 1:** Time to treatment-seeking by caretakers of children under-five with diarrhea in Uganda (*N* = 745)

Interval (Days)	Total Caretakers at time(t)	Caretakers that Sought Treatment at time(t)	The Probability of seeking treatment at time(t)	The Probability of not seeking treatment at time (t)
0–2	745	250	0.337	0.664
2–4	495	295	0.596	0.268
4–6	200	84	0.420	0.156
6–8	116	84	0.724	0.043
8–10	32	6	0.188	0.035
10–12	26	2	0.077	0.032
12–14	24	2	0.083	0.030
14–16	22	19	0.864	0.004
20–22	3	1	0.333	0.003
28–30	2	1	0.500	0.001
30–32	1	1	1.000	0.000

**Fig. 1:**
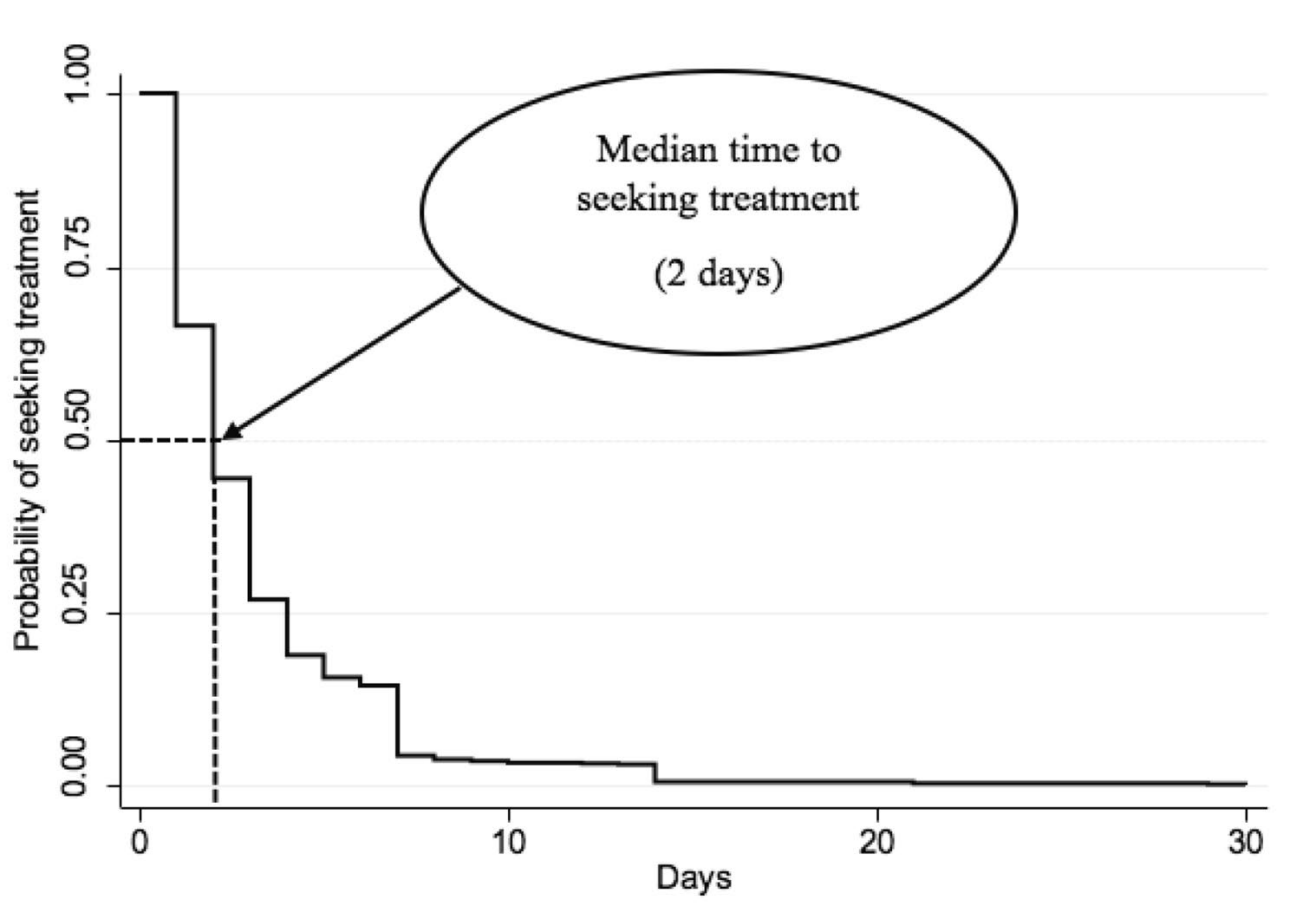
Kaplan meier survival estimates for time to seeking treatment *Source: authors computation*

Fig. 1 shows that the median time to seeking treatment was 2 days, which implies that caretakers among children under five with diarrhea spent on average 2 days before seeking treatment.

### Differentials in survival times

Table 2 presents the distribution of time spent seeking treatment of caretakers among children under five with diarrhea based on socio-economic and structural characteristics among the study participants. The survey involved a total of 745 caretakers of children under five with diarrhea. The results show that 38% children aged between 6 and 11 months and approximately 14% having infants less than 6 months old. Gender-wise, about 53% of the children were male, and 47% were female. The majority (53%) of caretakers were aged between 17 and 26 years, with only a small fraction (about 1%) aged 47 years and above. Furthermore, Females comprised the majority of caregivers, accounting for 93%. Educationally, the majority (41%) had attained a primary level of education, while 5% had no formal education. Geographically, approximately 71% of the participants resided in urban areas, and the majority (85%) were married. The distribution of caretakers by regions was as follows: 24% from the Northern region, 21% from the Eastern region, 28% from the Western region, and 27% from the Central region. Regarding wealth quintile, the majority (21%) were from poorest quintile, and 38% were engaged in business occupations. Health facility utilization showed that 55% of participants sought care from government facilities, while private facilities were less frequently utilized. The results also show that nearly (84%) had to travel 0–5 km to reach the nearest health facility.

**Table 2:** Differentials in time to seeking treatment by socio-economic and structural Factors

Covariates	*N*	Percentage distribution	Log-rank 𝑐h𝑖2, 𝑝
**Age of child (months)**			
< 6	102	13.7	
6–11	193	25.9	
12–23	283	37.9	9.09
> 24	167	22.4	(*p* = 0.028)
**Age of caretaker**			
17–26	395	53.0	
27–36	292	39.2	
37–46	48	6.4	2.88
>=47	10	1.3	(*p* = 0.410)
**Gender of child**			
Female	344	46.8	3.89
Male	391	53.2	(*p* = 0.049)
**Gender of caretaker**			
Female	694	93.2	(1.69)
Male	51	6.8	(*p* = 0.194)
**Education level of caretaker**			
No education	36	4.8	
Primary level	302	40.5	
Secondary level	289	38.8	8.72
Higher/Tertiary level	118	15.8	(*p* = 0.033)
**Residence**			
Rural	218	29.3	2.86
Urban	527	70.7	(*p* = 0.091)
**Marital Status**			
Single(unmarried)	64	8.6	
Married	633	85.0	1.20
Others	48	6.4	(*p* = 0.548)
**Region**			
Northern	181	24.3	
Eastern	159	21.3	
Western	205	27.5	74.31
Central	200	26.8	(*p* = 0.000)
**Wealth Quintile**			
Poorest	153	20.6	
Poor	143	19.3	
Medium	149	20.0	
Rich	146	19.7	12.79
Richest	151	20.4	(*p* = 0.012)
**Occupation**			
Agriculture	86	11.8	
Business	273	37.5	
Formal employment	167	22.9	4.91
Others	203	27.8	(*p* = 0.178)
**Health Facility**			
Government	410	55.0	1.70
Private	335	45.0	(*p* = 0.192)
**Distance to Health facility (km)**			
0–5	625	83.9	9.67
> 5	120	16.1	(*p* = 0.002)
All	745	100	

Differentials were assessed based on socio-economic and structural factors using the log-rank test for all categorical variables. This assessment aimed to determine which variables should be considered for multivariate analysis. The results of the factors considered for multivariate analysis revealed significant associations with the time spent seeking treatment among caretakers of children under five with diarrhea. Age of the child exhibited a statistically significant relationship (*p* = 0.028), indicating that different age groups may have distinct patterns in seeking treatment. Similarly, the gender of the child showed significance (*p* = 0.049), suggesting gender-specific differences in treatment-seeking behavior. Education level of the caretaker also played a significant role (*p* = 0.033), indicating variations in treatment-seeking tendencies based on the caretaker’s education. Geographically, the region of residence showed a highly significant association (*p* = 0.000), emphasizing the influence of geographical location on treatment-seeking timing. Additionally, wealth quintile demonstrated significance (*p* = 0.012), indicating the impact of socio-economic status on treatment-seeking behavior. Finally, distance to the health facility was significantly associated (*p* = 0.002), highlighting the influence of proximity to healthcare facilities on treatment-seeking decisions.

### Risk factors for time to seeking diarrhea treatment among children under five in Uganda

To identify the net effect of each independent factor on the time to seeking treatment, a multilevel proportional hazards model was employed, utilizing the risk factors identified in the bivariate analysis. In this context, all significant independent factors at the bivariate level were incorporated into the model as indicated in Table 3. These factors include the age of the child, gender of the child, education level of the caretaker, region, wealth quintile, and distance to the health facility. We found that caretakers with male children were more likely to delay in seeking treatment compared to those with female children (HR = 0.82; 95% CI = 0.71–0.95, *p* = 0.010). The hazard ratio (HR = 0.82) decreases among caretakers with male children implying longer time to seeking treatment. Furthermore, caretakers in the richest wealth quintile were more likely to seek treatment for their children early compared to those in the poorest quintile (HR = 1.37; 95% CI = 1.05–1.78, *p* = 0.022). The hazard ratio (HR = 1.37) increases among caretakers belonging to the richest wealth quintile indicating shorter time to seeking treatment. Regarding distance, caretakers living in more than 5 km from the health facility were more likely to delay in seeking treatment compared to those residing between 0 and 5 km to the health facility (HR = 0.68; 95% CI = 0.56–0.84, *p* = 0.000). The hazard ratio (HR = 0.68) decreases implying longer time to seeking treatment for caretakers living in more than 5 km from the heath facility. The estimated frailty variance was 0.13, indicating heterogeneity of treatment seeking time across the four regions in Uganda. Regarding the diagnostic test, the Akaike Information Criterion (AIC) of the Weibull model had the lowest value at 1783.951, in contrast to the AIC of the exponential model, which stood at 3112.541. Therefore, we reported and based our research findings on the Weibull model due to its lower AIC.

**Table 3:** Factors associated with time to treatment seeking by caretakers of children under-five with diarrhea in Uganda

Covariates	Hazards Ratio(HR)	*P*-Value	95% CI
**Age of child (months)**			
< 6 *(Ref.)*	1.00	-	-
6–11	1.01	0.948	0.79–1.29
12–23	1.15	0.251	0.91–1.44
> 24	1.25	0.084	0.97–1.60
**Gender of child**			
Female *(Ref.)*	1.00	-	-
Male	0.82	**0.010**	0.71–0.95
**Education level of caretaker**			
No education *(Ref.)*	1.00	-	-
Primary level	0.87	0.436	0.61–1.24
Secondary level	0.87	0.474	0.60–1.27
Higher/Tertiary level	1.10	0.644	0.73–1.66
**Wealth Quintile**			
Poorest *(Ref.)*	1.00	-	-
Poor	1.22	0.123	0.95–1.56
Medium	1.03	0.832	0.80–1.32
Rich	1.01	0.957	0.77–1.31
Richest	1.37	**0.022**	1.05–1.78
**Distance to Health facility (km)**			
0–5 (*Ref.)*	1.00	-	-
> 5	0.68	**0.000**	0.56–0.84
**Region** var(_cons)	0.13		0.03–0.55

(Ref.) = reference category, CI = confidence interval

## Discussion

This study aimed to explore the Time to Treatment-Seeking by Caretakers of Children Under-Five with Diarrhea and associated factors in Uganda using a Multilevel Proportional Hazards Model. The results of the study revealed that the median time to seek treatment was 2 days (range, 1–30 days) from the onset of diarrhea. As for the estimated frailty variance was 0.13, indicating heterogeneity across regions. Gender of the child, wealth quintile and distance to the health facility were the factors associated with time to seeking treatment by caretakers of children under five with diarrhea in Uganda.

Specifically, caretakers with male children were more likely to delay in seeking treatment compared to those with female children. This finding contrasts with the results of Sarker et al. (2016), who reported that male children were 2.09 times more likely to receive care than female children. However, this disparity can be explained by the mothers’ level of education. Educated women are better equipped to break away from traditional practices and utilize modern means to safeguard their children’s health. They can also make independent decisions regarding their children’s health, leading to better health outcomes for children, regardless of their gender [12].

Furthermore, caretakers in households belonging to the richest category were more likely to seek treatment for their children earlier than those in the poorest category. These findings align with previous studies [4, 7], where it was observed that families with lower income were less likely to seek timely healthcare due to higher expenditures and an inability to afford medical costs. The reason might be lower-income households often turn to unqualified or traditional healthcare providers for their children’s medical needs because of the lower cost, easy accessibility, and familiarity of these services in their areas [12]. This could also be due to the fact that economically disadvantaged households typically seek medical care for their children based on the perceived severity of illness, unlike wealthier households where regular medical checkups are scheduled.

Lastly, caretakers living in more than 5 km from the health facility were more likely to delay in seeking treatment compared to those residing between 0 and 5 km to the health facility. These findings align with the results of Kassile et al. (2014), who found that children living at a distance of > 5 km from the nearest health facility were twice as likely to delay seeking medical care compared to those living closer. This variation can be attributed to the distance from suitable healthcare facilities near their residence, potentially increasing out-of-pocket expenses for transportation and further discouraging caregivers from seeking early treatment [13].

Our present study has some limitations. The study did not employ a mixed methods analysis, incorporating both qualitative and quantitative approaches, which could have offered a more comprehensive understanding of treatment-seeking behaviors. In addition, the DOVE study’s utilization of cross-sectional data warrants careful consideration. Although the reported factors are associated with the outcome, caution must be exercised in assuming causality. Future research endeavors, particularly longitudinal studies, are needed to ascertain causal relationships and explore temporal variations in healthcare-seeking behaviors.

## Conclusion

The study showed that the median Time to Treatment-Seeking by Caretakers of Children Under-Five with Diarrhea in Uganda was 2 days. There are delays in seeking diarrhea treatment in Uganda because two days are enough to claim a life after dehydration. In addition, gender of the child, wealth quintile, and distance to the health facility were the factors associated with Time to Treatment-Seeking by Caretakers of Children Under-Five with Diarrhea in Uganda. Specifically, Caretakers in households belonging richest category were more likely to seek treatment for their children earlier than those belonging to the poorest category. However, caretakers with male children and those living in more than 5 km from the health facility were more likely to delay in seeking treatment. The policymakers should pay attention to formulate effective intervention to sensitize caregivers on the importance of early treatment-seeking behavior to avoid severe malnutrition caused by diarrhea. Community awareness program should also be encouraged particularly in areas of more than 5 km from the health facility to make people aware of the necessity to take prompt action to seek care in the early stage.

## Data Availability

The datasets used and/or analyzed during the current study available from the corresponding author on reasonable request.

## Abbreviations

AIC: Akaike Information Criterion
DOVE: Decade of Vaccination Economics
HC: Health Centers
HH: Household
HR: Hazard Ratio
ICF: International Classification of Functioning, Disability and Health
KM: Kilometers
MDG: Millennium Development Goal
ORS: Oral Rehydration Solution
OR: Odds Ratio
PCA: Principle Component Analysis
*p*-value/p: Probability Value
U5: Under five years
UBOS: Uganda Bureau of Statistics
USAID: United States Agency for International Development
UN: United Nations
